# Age-related differences in the bone marrow stem cell niche generate specialized microenvironments for the distinct regulation of normal hematopoietic and leukemia stem cells

**DOI:** 10.1038/s41598-018-36999-5

**Published:** 2019-01-30

**Authors:** Ga-Young Lee, Seon-Yeong Jeong, Hae-Ri Lee, Il-Hoan Oh

**Affiliations:** 1 Catholic High-Performance Cell Therapy Center and Department of Medical Lifescience, The Catholic University of Korea, College of Medicine, Seoul, 137-701 Korea; 2 Department of Medical Lifescience, The Catholic University of Korea, College of Medicine, Seoul, 137-701 Korea

## Abstract

The bone marrow (BM) microenvironment serves as a stem cell niche regulating the *in vivo* cell fate of normal hematopoietic stem cells (HSC) as well as leukemia stem cells (LSCs). Accumulating studies have indicated that the regeneration of normal HSCs and the process of leukemogenesis change with advancing age. However, the role of microenvironmental factors in these age-related effects are unclear. Here, we compared the stem cell niche in neonatal and adult BM to investigate potential differences in their microenvironmental regulation of both normal and leukemic stem cells. We found that the mesenchymal niche in neonatal BM, compared to adult BM, was characterized by a higher frequency of primitive subsets of mesenchymal stroma expressing both platelet-derived growth factor receptor and Sca-1, and higher expression levels of the niche cross-talk molecules, Jagged-1 and CXCL-12. Accordingly, normal HSCs transplanted into neonatal mice exhibited higher levels of regeneration in BM, with no difference in homing efficiency or splenic engraftment compared to adult BM. In contrast, *in vivo* self-renewal of LSCs was higher in adult BM than in neonatal BM, with increased frequencies of leukemia-initiating cells as well as higher lympho-myeloid differentiation potential towards biphenotypic leukemic cells. These differences in LSC self-renewal capacity between neonates and adults was abrogated by switching of recipients, confirming their microenvironmental origin. Our study provides insight into the differences in leukemic diseases observed in childhood and adults, and is important for interpretation of many transplantation studies involving neonatal animal models.

## Introduction

Hematopoietic stem cells (HSCs) are rare subsets of hematopoietic cells that are responsible for life-long production of all blood cells lineages, and for the reconstitution of bone marrow (BM) after transplantation into myeloablated recipients^1,2^. Studies have shown that the bone marrow (BM) microenvironment plays a key role in regulating the regenerative activity of HSCs by causing their switch between a dormant and active state^3–5^, and controlling the self-renewal^6,7^, quiescence^8–10^, and mobilization^11^ of HSCs. The niche cells express molecules such as Jagged-1^7,12^, CXCL-12^13–15^, and angiopoietin-1^16^ that cross-talk with HSCs and exert a microenvironmental influence on hematopoiesis. Thus, the stem cell niche is a major parameter that controls the regeneration of transplanted HSCs and thereby maintains blood homeostasis.

The stem cell niche also serves as a primary engraftment site for leukemic stem cells (LSCs) to initiate leukemogenesis, i.e., LSCs compete with normal HSCs during their engraftment into the BM niche^17,18^. The BM niche is reprogrammed under leukemic conditions into a degenerative leukemic niche that selectively supports leukemic cells while suppressing normal HSC activity^19–21^. This leads to the dominance of leukemic cells over normal HSCs^22,23^. Thus, the microenvironment of the BM exerts a pivotal regulatory influence on the proliferation and engraftment of normal HSCs as well as of leukemic stem cells (LSCs).

Recently, studies have shown that the microenvironment of HSCs can change with ontological stage of hematopoietic development exhibiting differences in niche composition^24–27^. For example, the development of BM from fetal, through neonatal, to adults is associated with changes in the expression of extracellular matrix (ECM) markers including tenascin or osteopontin^28,29^. Similarly, stromal cells in the niche exhibit changes in their proliferative capacity and differentiation potential with changes to the physical properties and chemical composition of the ECM^25,30,31^. Of note, HSCs at different ontological stages also exhibit distinct hematopoietic features related to the cell cycle, proliferation potential, and long-term hematopoietic functions^32^. Moreover, human leukemic diseases exhibit distinct clinical spectrums and incidence, and differences in their response to treatment between children and other age groups^33–35^. However, it is unclear whether differences in the niche influence these age-related differences in the regenerative and leukemogenic activities of normal HSC and LSCs.

The ontological changes in the microenvironment are also important for many *in vivo* studies, since transplantation into the neonate BM niche is frequently employed as a model to explore the engraftment kinetics of HSCs and subsequent reconstitution of the immune system^36,37^, because they achieve a higher level of engraftment than in adult models^38^. Similarly, neonatal transplantation is also frequently employed to analyze the leukemogenic process of LSCs, metastasis^39^ and their response to chemotherapy^17^. However, despite this wide-spread use of the neonatal mice transplantation model, the specific influence of the neonatal BM microenvironment on HSCs or LSCs, compared to those of adult BM, has not been well established.

Therefore, in this study, we compared the characteristic changes of the microenvironment in neonate and adult BM, and examined their functional influence on normal HSCs and LSCs. Our study reveals a unique functional influence of the neonatal BM microenvironment distinct from the adult BM, providing important insight into the differences in hematological malignancies between childhood and adulthood, as well as considerations for the many *in-vivo* studies utilizing the neonatal model.

## Results

**Figure 1:**
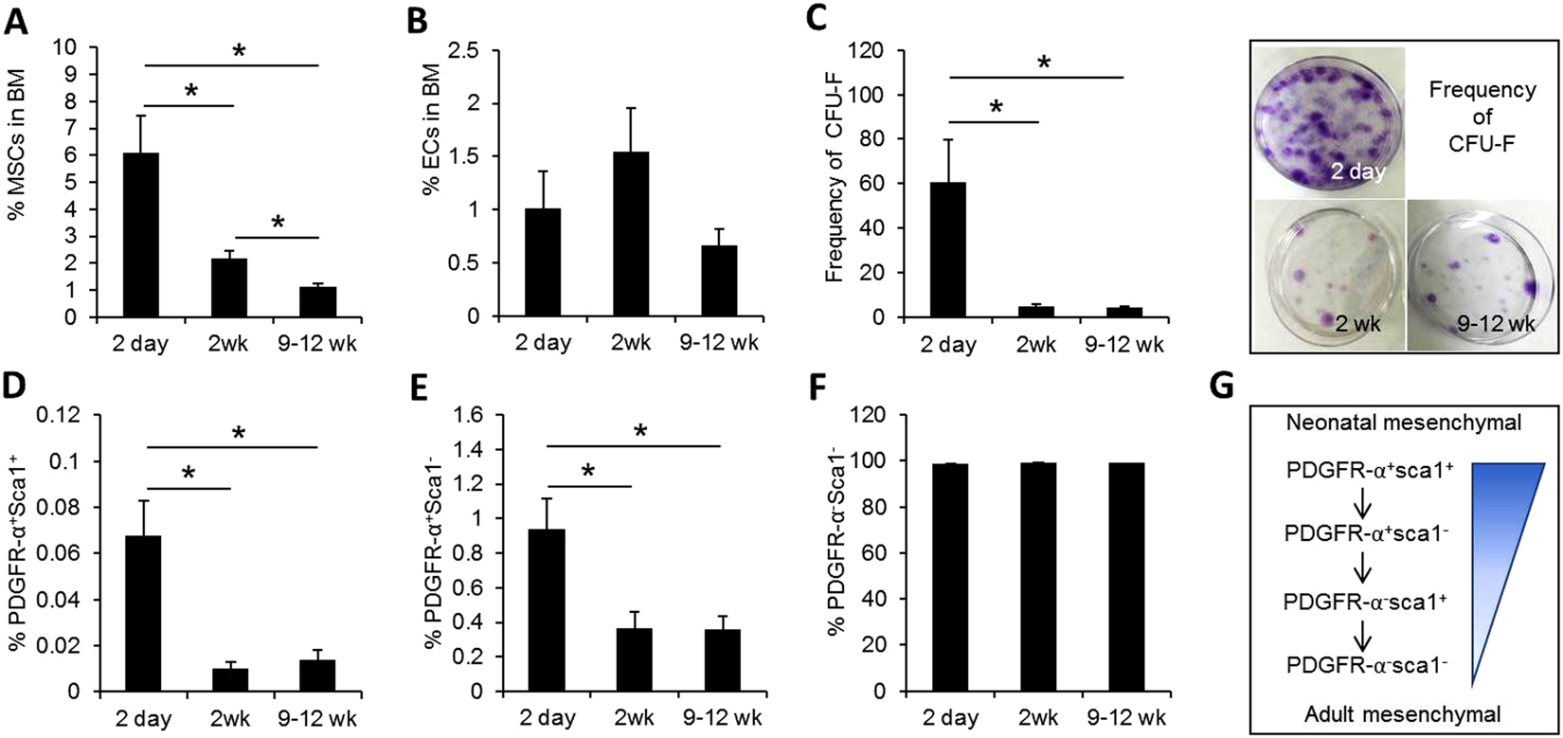
Age-related differences in stromal cell composition of BM. (**A**,**B**) Cellular composition of stromal cells in BM were compared for mice at indicated ages. Shown are the % of mesenchymal stroma (**A**) and endothelial cells (**B**) at 2 days, 2 weeks, and 9–12 weeks after birth (Mean ± SEM, n = 5 for 2 days, n = 10 for 2 weeks, n = 16 for 9–12 weeks, from 7 expts). (**C**) Comparisons of frequencies of clonogenic mesenchymal cells (CFU-F) in neonate and adult BM. Shown are the numbers of CFU-F obtained by plating 5 × 10^6^ BMCs and representative photographs of colonies visualized by crystal violet staining (Mean ± SEM, n = 3 for day2, n = 8 for 2 weeks, n = 13 for 9–12 weeks, from 6 expts, *p < 0.05). (**D**–**G**) Difference in the composition of mesenchymal subsets among total mesenchymal stroma in BM. Shown are % of most primitive PDGFR + Sca-1+ (**D**), intermediate PDGFR + Sca1− (**E**), and mature PDGFR-Sca1− (**F**), subsets among the total mesenchymal population (CD45-Ter119-CD31−) (Mean ± SEM, n = 5 for day 2, n = 10 for 2 weeks, n = 16 for 9–12 weeks, from 7 expts). (**G**) Schematic illustration of hierarchical organization of MSC subsets.

To explore the potential differences in the microenvironment of neonatal and adult BM, we first examined the difference in BM stromal cells between neonate (postnatal day 2) and adult (9–12 weeks) including mesenchymal and endothelial cells (MSCs and ECs, respectively), which are the major stromal cell components comprising the BM niche. We found that the proportions of mesenchymal stromal cells (CD45-Ter119-CD31−) in the BM was highest in the neonate (postnatal day 2), and decreased thereafter to adult levels by 2 weeks after birth (Fig. 1A). In contrast, no significant changes were observed in the frequency of endothelial cells (EC: CD45-Ter119-CD31+) between the age groups (Fig. 1B). Thus, quantitative differences in MSCs rather than in ECs are likely contributing to the difference in the BM microenvironment at different ages. To further examine the difference in cellular composition of the mesenchymal niche we analyzed the MSC subpopulations in neonate and adult BM. We found that the neonatal BM exhibited higher frequencies of clonogenic MSCs that can form colony-forming unit fibroblasts (CFU-F) (Fig. 1C). Phenotypically, neonate BM exhibited higher frequencies of primitive mesenchymal subsets characterized by the expression of PDGFR and Sca-1 (PDGFR + Sca-1+)^40^, than other age groups in consistence to the higher frequency of colony forming units in neonatal BM. However, the differences in BM mesenchymal populations became less profound for MSCs at more differentiated stages: moderate differences for intermediate stage MSCs (PDGFR + Sca-1−), and no significant difference for differentiated subsets (PDGFR-Sca-1−) (Fig. 1D–F). Thus, the composition of the *in-vivo* mesenchymal niche changes with age, exhibiting a hierarchical organization of primitive stage MSC subsets with respect to ontological stage (Fig. 1G).

In contrast, culture established MSCs from neonate or adult BM exhibited comparable cell size, morphology, surface phenotypes, population doubling times and cell cycling during *in-vitro* culture (Supplemental Fig. 1A–E). Similarly, cultured neonatal MSCs exhibited comparable multi-lineage differentiation, albeit higher adipogenic, but lower osteogenic differentiation than adult MSCs (Supplemental Fig. 1F,G).

**Figure 2:**
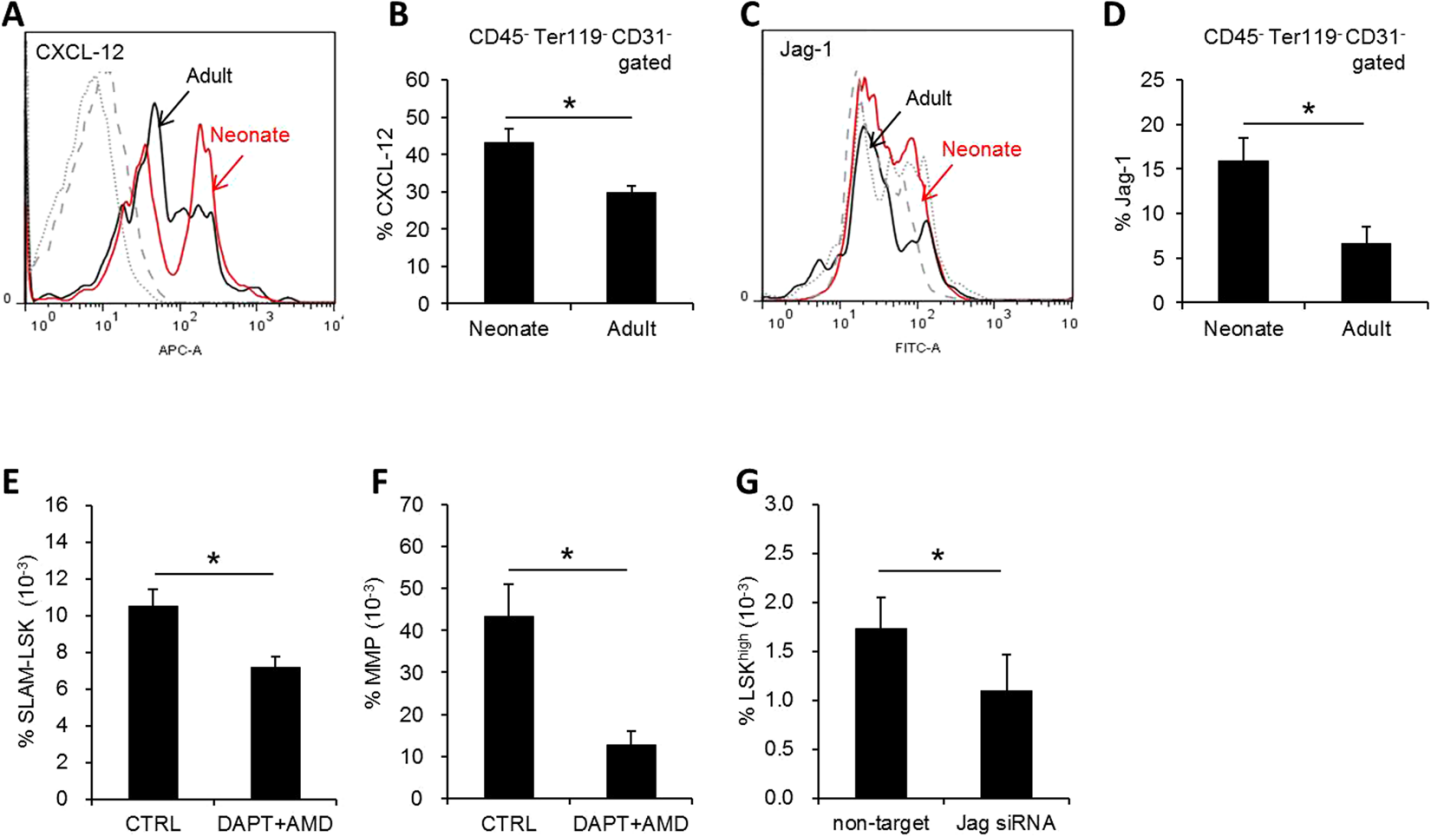
Difference in the expression levels of niche cross-talk molecules in mesenchymal stroma of neonate and adult BM. (**A**–**D**) Adult and neonatal (postnatal day2) BM stromal cells were compared for expression levels of CXCL-12 and Jagged-1. Shown are the representative flow cytometry profiles for intracellular staining and quantification of CXCL-12 (**A**,**B**) and Jagged-1 (**C**,**D**) in mesenchymal stroma (CD45-Ter119-CD31−) of BM (Mean ± SEM, n = 7 for neonate, n = 6 for adult, from 2expts). (**E**,**F**) Functional influence of niche cross-talk molecules on *in-vivo* hematopoietic activity. Mice were injected with chemical inhibitors of CXCL-12 (AMD3100) and notch ligand (DAPT) 4 times every two days and 2weeks later resulting changes of BM in the % of HSCs (SLAM-LSK) or multiple progenitors (LSKCD150-CD41/48−) were analyzed (Mean ± SEM, n = 6-7, 2expts, *p < 0.05). (**G**) Effects of Jagged-1 knock down in MSCs on *in-vitro* self-renwal of hematopoietic progenitor cells. MSCs transfected with siRNA against Jagged-1 or non-target RNA were co-cultured with progenitor-enriched hematopoietic cells. Shown are the % of LSK^high^ population in hematopoietic cells after 5 days of co-culture with each indicated MSCs (Mean ± SEM, n = 6, from 2 expts, *p < 0.05).

Next, to compare the microenvironmental function of mesenchymal cells in neonatal and adult BM, we compared expression of extracellular signaling molecules, Jagged-1 and CXCL-12, in BM mesenchymal cell population, the two molecules in BM niche that serve as cross-talk signals to regulate HSC self-renewal^12,41^. We found that MSCs in neonatal BM expressed significantly higher levels of CXCL-12 and Jagged-1, compared to MSCs from adult BM (Fig. 2A–D). We also found that mice treated with inhibitors of CXCL-12 and Jagged-1 (AMD3100 and DAPT, respectively) caused significant decrease of hematopoietic progenitor pool in BM and knock down of Jagged-1 decreased *in-vitro* self-renewal of the progenitor cells (Fig. 2E–G), in consistence to previous observations^12,41,42^. Taken together, these results show that the neonatal BM microenvironment is characterized by higher proportions of primitive MSC subsets with increased expression of niche cross-talk molecules that can support higher self-renewal of HSCs than in adult BM.

**Figure 3:**
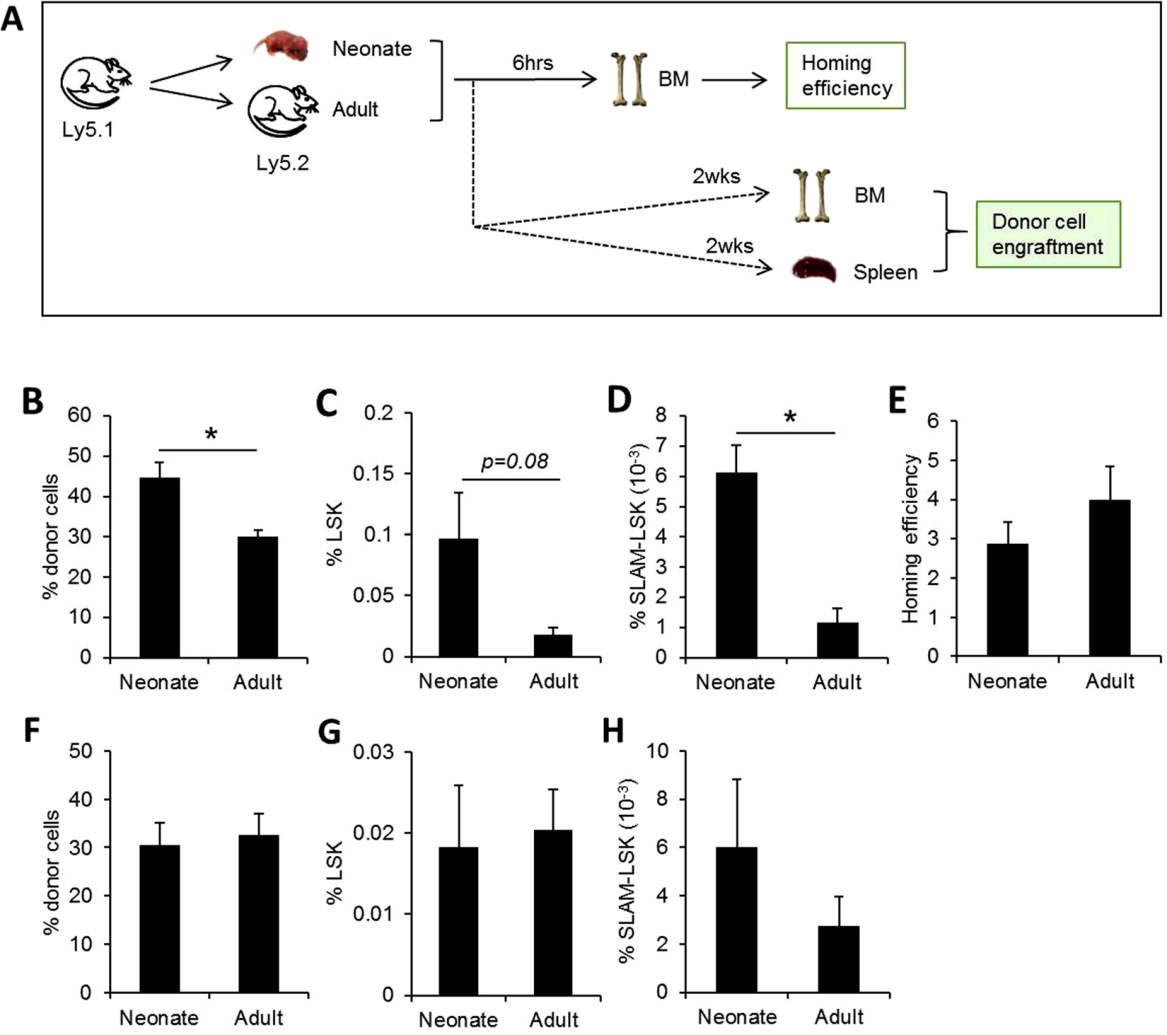
*In vivo* repopulation of normal hematopoietic cells in neonatal and adult recipients. (**A**) Schematic illustration of experimental scheme. Bone marrow cells (BMCs) from the donor mice (Ly5.1) were transplanted into irradiated neonate (postnatal 2 day) or adult (9–12 weeks) recipients (Ly5.2). Homing efficiency and engraftment of transplanted cells were analyzed 6 hours and 2 weeks after transplantation, respectively. (**B**–**D**) Engraftment of donor-derived cells in BM of neonate and adult mice were compared. Shown are the mean ± SEM of % engraftment of donor-derived cells (**B**), and % hematopoietic stem cells (HSCs) among donor cells defined by LSK and SLAM-LSK (CD150 + 41-48-Lin-Sca-1 + c-Kit+) (**C**,**D**) (n = 5, from 2 expts, *p < 0.05). (**E**). Homing efficiency of transplanted donor BMCs into neonate and adult BM. % donor-derived cells in BM of neonate and adult mice 6 hours after transplantation are shown (Mean ± SEM, n = 3 for neonate, n = 4 for adult mice, 2expts). (**F**–**H**) Splenic engraftment of normal HSCs. % engraftment of donor-derived hematopoietic cells (**F**) and % of HSCs (LSK or SLAM-LSK) among donor cells (**G**,**H**) in spleen are shown. (Mean ± SEM, n = 7, from 3 expts).

To further confirm the observation, we next compared the engraftment kinetics of normal HSCs in recipients in neonatal and adult recipients relative to their homing efficiency (Fig. 3A). When normal BM cells (BMCs) were transplanted into irradiated neonatal or adult recipient mice, neonatal BM exhibited significantly higher engraftments of donor cells than adult BM (Fig. 3B). This increased engraftment was not related to differences in homing efficiency but was characterized by increased regeneration of HSCs, as evidenced by increased frequencies and numbers of primitive HSC subsets (LSK: Lin-Sca-1 + c-Kit+ or SLAM-LSK: CD150 + CD41-CD48-Lin-Sca-1 + c-Kit+) among engrafted donor-origin cells (Fig. 3C,D and Supplemental Fig. 2) without significant difference in homing efficiency (Fig. 3E). In contrast, no significant difference was seen for engraftment of donor cells or frequencies of HSCs among cells engrafted in spleen (Fig. 3F–H). These results show that the neonatal BM niche provides a microenvironment that promotes greater self-renewal of HSCs than the adult BM niche.

**Figure 4:**
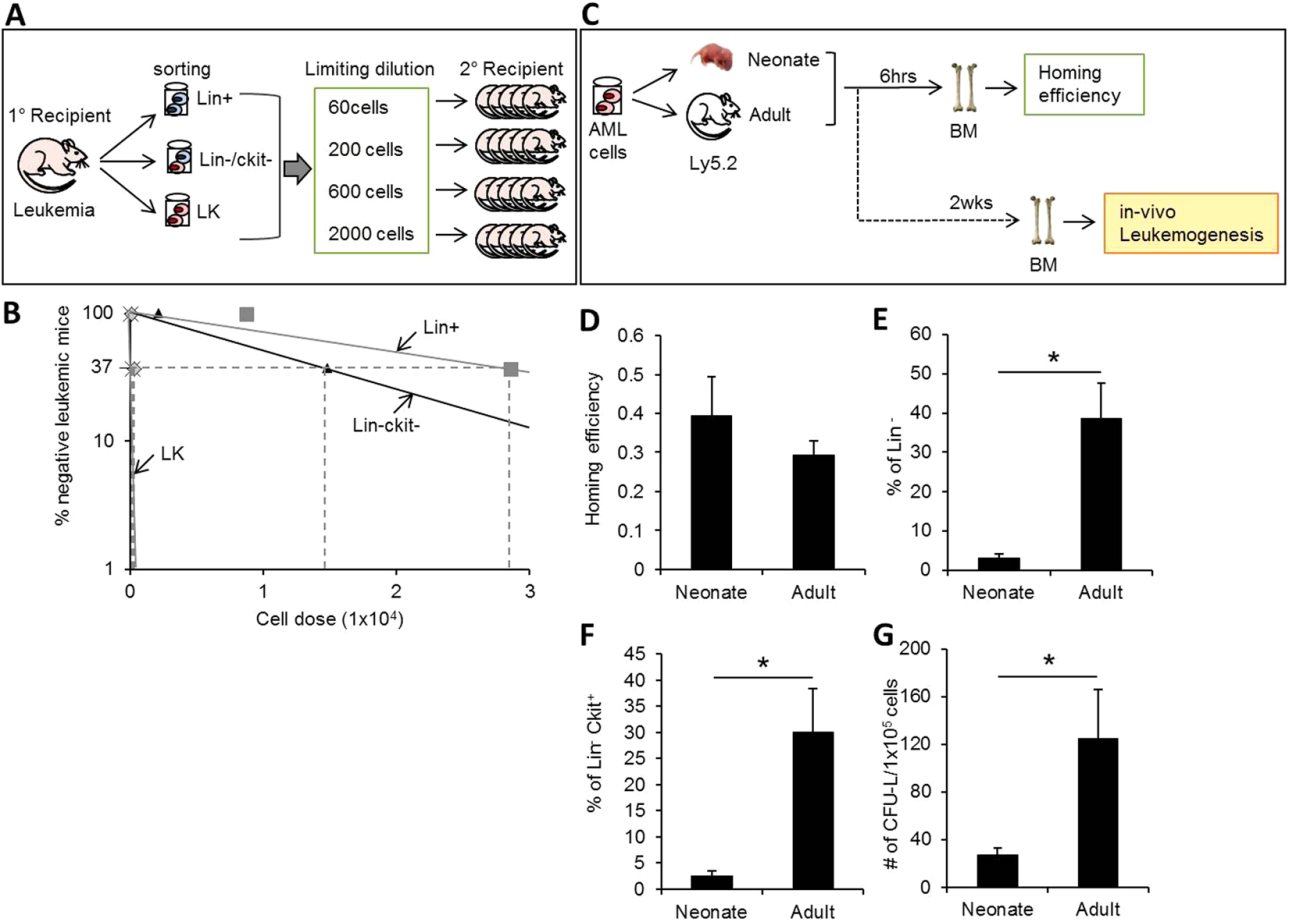
Comparison of *in vivo* leukemogenic activity between neonate and adult BM. (**A**) Schematic illustration of the experimental design for Identification of leukemia-initiating cell subpopulations in acute myeloid leukemia (AML) by limiting dilution analysis. 5-FU BMCs were transduced with retrovirus encoding the MN1 oncogene to induce leukemia. Two weeks after transplanting AML cells into recipient mice, the heterogenous leukemic cell population generated in the BM of leukemic mice were sort purified by phenotype and subjected to limiting dilution analysis (LDA) into secondary recipients to measure the frequency of leukemia-initiating cells (LIC) at post-transplantation 2 weeks. (**B**) Frequency of LIC in each purified subset of leukemia cells in LDA plots calculated by Poisson’s statics. (**C**) Schematic illustration of experimental design for comparing leukemogenic activity. Acute myeloid leukemia (AML) cells induced by MN1 were transplanted into mice and their homing and leukemic engraftment was analyzed at 6 hrs (homing) and 2 weeks (engraftment) after transplantation. (**D**) Comparisons of homing efficiency into BM. Shown are the % of leukemic cells in recipient BMs (Mean ± SEM, n = 4 for each group, from 2expts). (**E**,**F**). Analysis of *in vivo* generation of LSCs in leukemic recipient mice. Shown are the % engraftment of each subset of leukemic cells in mice (Mean ± SEM, n = 13 for neonatal group, n = 6 for adult group, from 5 expts). (**G**). Numbers of clonogenic leukemic cells (CFU-L) from 1 × 10^5^ of leukemic cells (GFP+) engrafted in neonate and adult BMs (Mean ± SEM, n = 15 for neonate, n = 8 for adult, from 3 expts, *p < 0.05).

Since the BM niche also serves as a microenvironment for leukemic stem cells as well as for normal HSCs^17,19–21^, we next examined their influence on *in vivo* self-renewal of leukemic stem cells (LSCs). For this, we employed a murine leukemia model induced by exogeneous expression of MN1^43^. Transduction of MN1 into progenitor-enriched BMCs led to their transformation into acute myeloid leukemia cells (AML cells). Subsequent transplantation of these AML cells into recipients led to *in-vivo* leukemogenesis (Supplemental Fig. 3). To identify the LSC subpopulation among the MN1 leukemic cell population engrafted in these mice, three heterogeneous leukemic subpopulations (i.e., Lin+, Lin-c-Kit−, and Lin-c-Kit+ cells) generated during *in-vivo* leukemogenesis were sort purified from primary recipient and subjected to secondary transplantation to compare *in-vivo* leukemogenic activity (Fig. 4A). We found that leukemogenic activity (i.e., the frequency of leukemia initiating cells) was significantly enriched in Lin-c-Kit+ (LK) cells, compared to the Lin+ or Lin-c-Kit− populations (Fig. 4B), thus identifying LK cells as leukemia-initiating cell population in this model.

Based upon the findings, we examined the *in vivo* self-renewal capacity of these LSCs in the BM of adult and neonate mice that had been transplanted with MN1 leukemic cells (Fig. 4C). The homing efficiency of the leukemic cells to the BM was comparable between neonates and adults (Fig. 4D). However, the frequency of the more primitive subpopulations, including the Lin- cells or LK cells, among the BM engrafted leukemic cells was significantly higher in adult recipients than in neonates (Fig. 4E,F and Supplemental Fig. 4). Similarly, the frequency of clonogenic leukemic cells (CFU-L) was also significantly higher when transplanted into adults than into neonates (Fig. 4G). These results indicate that the self-renewal capacity of LSCs is higher in adult than in neonate BM, which is in contrast to that of normal HSCs.

**Figure 5:**
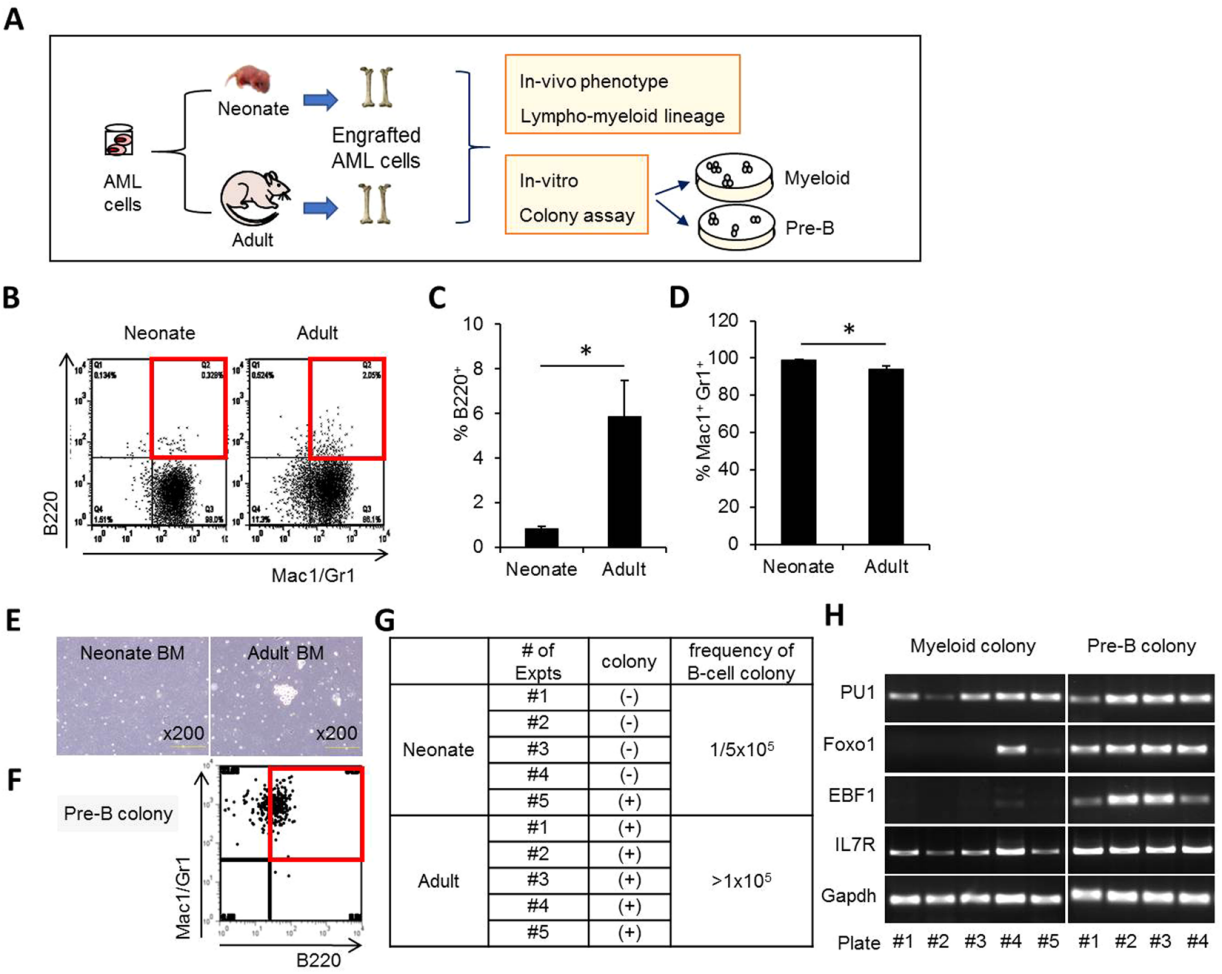
Multi-lineage potential of leukemic cells in adult BM. (**A**) Schematic illustration of experimental design. After transplanting adult or neonatal mice with leukemic cells. The leukemic cells (GFP+) engrafted in neonatal or adult BM were analyzed for phenotype by flow cytometry and plated for myeloid or pre-B-cell colony formation. (**B**) Representative flow cytometry plots for lineage distribution (myeloid: Mac-1/Gr-1+ vs. B-lymphoid: B220+/Mac-1/Gr-1+). (**C**,**D**) Percent of biphenotypic B-lymphoid (B220/Mac-1/Gr-1)) (**C**) and myeloid cells (Mac-1/Gr-1) (**D**) among engrafted GFP+ cells are shown (Mean ± SEM, n = 13 for neonatal group, n = 6 for adult group, from 5 expts). (**E**–**G**) B220+ colonies generated from BM leukemic cells engrafted in neonate and adult BM. Shown are representative photographs of colonies formed in pre-B-cell colony assay (**E**) and phenotypes of individual colonies analyzed by flow cytometry (**F**). (**G**) frequencies of positive B-lymphoid colonies from 1 × 10^5^ (GFP+) plated cells (2 expts, n = 5 for each group). (**H**) Expression of B-cell specific genes in colonies formed in pre-B-cell colony assay. The colonies generated in myeloid or pre-B-cell colony assay from BM engrafted leukemic cells were analyzed for indicated B-cell specific genes by RT-PCR. Note selective expression of B-cell specific genes in pre-B-cell colonies.

To further explore the different behavior of LSCs in neonate and adult recipients, we compared the differentiation phenotype of leukemic cells engrafted into neonate and adult BM (Fig. 5A). Leukemic cells engrafted into adult recipient mice were of a mostly myeloid lineage, but there was a significantly higher number of biphenotypic population that exhibited both B-lymphoid (B220+) and myeloid (Mac-1/Gr-1) markers than in those engrafted into neonatal BM (Fig. 5B–D). The increase in B-cell like phenotype of engrafted leukemic cells was also supported by the higher frequency of B220+ colonies in pre-B cell colony assay (CFU-preB) among engrafted leukemic cells (Fig. 5E–G) and their expression of B-cell specific transcription factors such as PU.1, Foxo1, EBF1, and IL-7 receptors^44–46^ (Fig. 5H). Therefore, taking previous studies indicating that the biphenotypic leukemic cells represent pluripotent type of leukemic cells^47–51^, these results showed that the primitive state of LSCs (i.e., higher self-renewal and multi-lineage potential) are better maintained in adult BM than in neonate BM.

**Figure 6:**
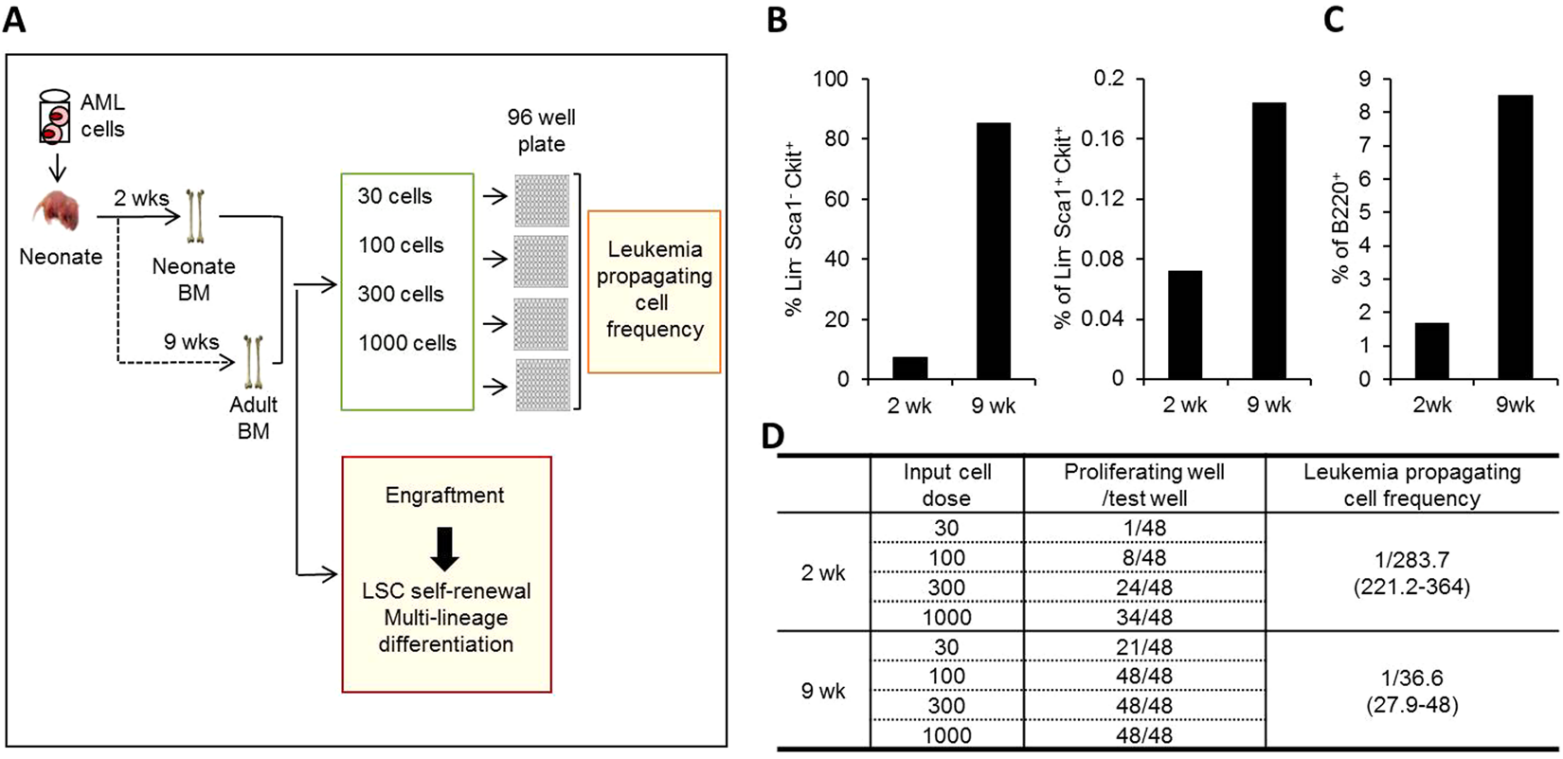
Time-lapse changes of leukemic cell with aging of BM microenvironment. (**A**) Schematic illustration of experimental design. Leukemic cells were transplanted into neonate and examined for multi-lineage differentiation and self-renewal of LSCs with aging of recipient BM at 2 weeks and 9 weeks after transplantation. (**B**,**C**) Changes in the frequencies of primitive leukemic population (Lin-Sca-1-c-Kit+ or Lin-Sca-1 + c-Kit+) (**B**) and B220+ leukemic cells (**C**). Shown are the mean % of each subset among BM engrafted leukemic cells (n = 2, from 1 expt). (**D**) Changes in frequency of leukemia-propagating cells determined by *in-vitro* limiting dilution assay.

**Figure 7:**
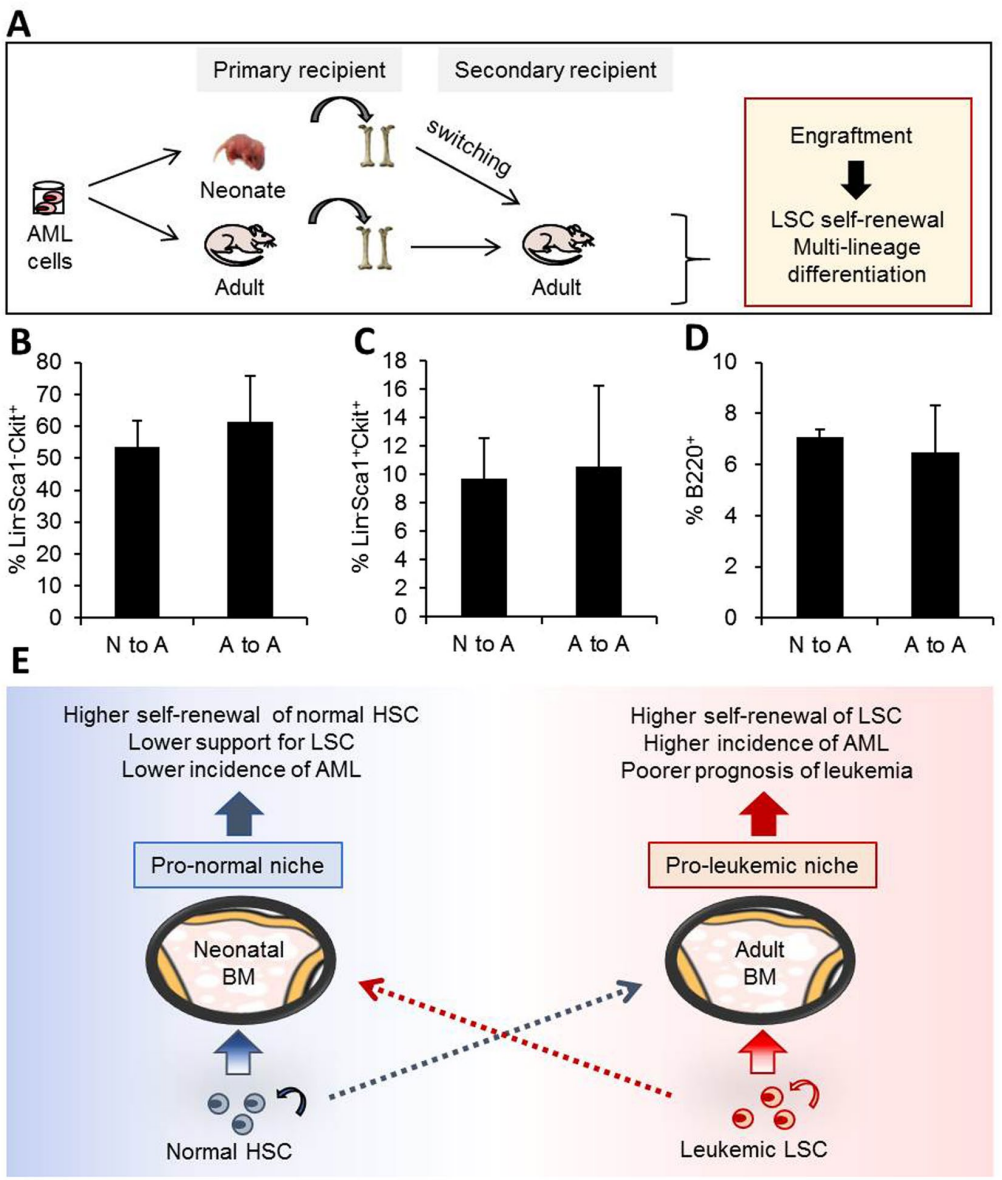
Microenvironmental origin of the ontological difference in leukemogenic activity. (**A**) Schematic illustration of experimental design. Leukemic cells were transplanted into neonate and adult mice. Two weeks after transplantation, the leukemic cells engrafted in primary recipients were subjected to switching transplantation into adult recipients to compare *in vivo* generation of LSCs and multi-lineage differentiation into B-cells (post-transplantation 2 weeks). (**B**–**D**) Percent of Lin-Sca-1-c-Kit+ (**B**), Lin-Sca-1 + c-Kit+ (**C**), and B220+ leukemic cells (**D**) among leukemic cells in switched secondary recipients are shown (Mean ± SEM, n = 4). (**E**) Schematic summary of the age-related differences in the neonatal BM microenvironment compared with the adult BM. The neonatal BM niche is characterized by higher frequencies of primitive mesenchymal subsets and higher levels of niche cross-talk. The neonatal niche provides a microenvironment for enhanced regeneration of normal HSCs, whereas adult BM supports enhanced regeneration of LSCs, which is consistent with the clinical observations of higher incidence of AML and poorer prognosis in adult patients compared to leukemia in childhood (boxed arrows indicate enhanced, dashed arrows suppressed engraftments).

Next, to further examine the role of the microenvironmental ages in the observed differences in LSC behavior, we examined the time-lapse changes of LSCs that had been transplanted into neonatal BM at the time points of 2weeks (neonatal stage) or 9 weeks (adult stage) after transplantation (Fig. 6A). We found that leukemic cells engrafted in neonate become adult type leukemia exhibiting higher frequency of primitive population (Fig. 6B) and B-lymphoid leukemic cells (Fig. 6C). Similarly, leukemic cells at 9 weeks after transplantation exhibited higher frequency of leukemia-propagating cells than those at 2 weeks after transplantation, as determined by *in-vitro* limiting dilution assay (Fig. 6D). To directly examine these findings, we next performed a switching transplantation experiment with secondary recipient mice. For this, leukemic cells engrafted into neonate or adult BM were harvested and both were second transplanted into the adult recipient mice to see if the differences were dependent on the recipients (Fig. 7A). The observed differences in LSCs between neonate and adult recipients were abrogated when these leukemic cells were transplanted into adult mice, i.e., there were comparable frequencies of LSCs (LK or LSK) among the engrafted leukemic cells (Fig. 7B,C), as well as comparable levels of B-lymphoid leukemic cells (Fig. 7D).

Together, these results confirm the microenvironmental origin of the LSC differences between the neonate and adult groups. Thus, our results show that LSCs undergo higher self-renewal in adult BM in a manner dependent on the microenvironment, and that adult and neonate BMs provide unique stem cell niches that distinctively support normal HSC and LSCs.

## Discussion

Different developmental stages have specific influences on developing cells. For hematopoiesis, HSCs are generated in different organs, including the aorta-gonad-mesonephros (AGM), fetal liver and bone marrow. HSCs at each developmental stage exhibit distinctive functional characteristics with respect to the cell cycle, response to cytokines, proliferation potential, and long-term hematopoietic function^32,52–54^. Accordingly, many hematopoietic functions, including myeloid and immune functions, become distinct across the fetal, neonatal and adult stages, which in turn become differentially vulnerable to hematological disorders such as bone marrow failure or hematological malignancies^53–56^. Interestingly, substantial evidence has revealed that the microenvironment for HSCs also changes during developmental maturation, i.e., differences in the structure of the extracellular matrix^25,30,31^ along with expression changes of ECM components in stromal cells from fetal, through neonatal to adult stages^28,29^. Notably, while these differences could potentially contribute to differences in hematopoietic function^57^, they could also exert a differential influence on the behavior of leukemia stem cells (LSCs) at different ages, since the BM microenvironment plays a pivotal role in regulating their self-renewal, survival, and chemoresistance^17,20,22,23^.

In our investigation of the ontological differences in the microenvironment, we found a characteristic and hierarchical difference in the composition of the mesenchymal niche between neonates and adults: neonate BM is significantly enriched with primitive subsets of MSCs (PDGFR + Sca-1+), and the neonatal MSCs expressed higher levels of niche cross-talk molecules shown to stimulate self-renewal of HSCs^12,20,41^. Consistent with these findings, the neonatal BM niche provided a more supportive microenvironment for self-renewal of normal HSCs than the adult BM.

Interestingly, this higher regeneration capacity of normal HSCs in neonatal BM was the opposite for leukemic stem cells, i.e., LSCs underwent greater self-renewal in adult BM than in neonatal BM. This was characterized by higher levels of leukemia initiating cells. Similarly, LSCs engrafted into adult BM exhibited a higher extent of biphenotypic (B-lymphoid and myeloid) as well as the myeloid lineage from myeloid-committed leukemic cells. Accumulating studies have reported subtypes of AML exhibit both myeloid and lymphoid markers in leukemic blast (biphenotypic leukemia). The studies also showed that these biphenotypic leukemia cells represent leukemic progenitors with stemness and multi-lineage potential capable of differentiating into both lymphoid and myeloid lineages exhibiting poor clinical prognosis^47–51^. Therefore, our finding for higher frequency of biphenotypic leukemic cells in adult BM than in neonate BM, together with higher self-renewal, further supports our model that LSCs in adult BM are maintained in a more primitive state than in neonatal BM. At present, the precise mechanisms underlying these differences in LSC characteristics between neonate and adult BM are not clear. One could speculate that there is a difference in the clonal heterogeneity of leukemic subpopulations and some selective engraftment of distinct leukemic cell subpopulations in neonate compared to adult BM. However, our findings shows that the leukemic cells engrafted in neonate become adult type with aging of the recipients with higher frequencies of primitive LSC populations and B-lymphoid leukemic cells. Similarly, our switching transplantation experiment show that leukemic cells engrafted into neonates, when transplanted into adult recipients, become like LSCs in adult BM, exhibiting comparable levels of LSC regeneration and B-lymphoid cell production. Thus, the difference between LSCs engrafted into neonate or adult BMs originates from the microenvironment.

The clinical significance of the distinct roles of the microenvironment of neonate and adult BM is not yet clear. However, it is notable that age is a major factor in leukemic disease with respect to disease progression and clinical prognosis^34^. Accordingly, the spectrum of hematological malignancies differs markedly between children and adults, with distinct biological characteristics of leukemic cells and their responses to treatment^33^. For example, acute myeloid leukemia occurs more frequently in elderly patients than in childhood or adolescence, and the prognosis becomes poorer with increasing age^34^. To date, the basis for these differences has been largely attributed to the difference in the molecular alterations in the blasts, as supported by recent genomic studies in Children’s Oncology Group (COG) AML trials analyzing 1,000 leukemias in children^58^. However, it is noteworthy that the prognosis in aged leukemia patients was poorer compared to children even in the absence of specific genetic aberrations or other known poor risk factors in the leukemic blasts^35^. This observation further implicates a substantial influence of microenvironmental factors on distinct leukemogenic process. Consistent with this, we recently showed that the BM microenvironment in AML patients influences their leukemogenic patterns, i.e., differences in the composition of BM mesenchymal stroma influences leukemogenesis and relapse in AML patients, serving as a parameter for clinical prognosis^20^. Therefore, given the differences in mesenchymal stromal cell composition between neonates and adults, it is possible that age-dependent changes in the BM microenvironment are a previously unrecognized factor for distinguishing the clinical spectrum of the disease, as well as disease progression between children and adult patients. Further studies are warranted to confirm the clinical significance of ontological differences in the microenvironment in leukemia patients.

Understanding the ontological differences in the BM microenvironment are also important when considering the current use of neonatal mice as a model to study the *in vivo* behavior of transplanted cells^59^, i.e., HSC transplantation into neonatal mice BM is frequently employed for various functional studies on hematopoiesis^38^, immune system development^36,37^, and leukemogenisis^17,39^. Therefore, care should be taken in the interpretation of findings from neonatal models and their extrapolation to adult models, taking that they have different influences on HSCs versus leukemic cells.

Our study demonstrates distinctive microenvironmental influences on the function of normal and leukemic stem cells that provide important insights into age-related changes in hematological disease, as well as a guide for experimental studies utilizing neonatal animal models.

## Materials and Methods

### Animals

C57BL/6J-Ly5.2 (BL6) mice or C57BL/6J-Pep3b-Ly5.1 (Pep3b) mice used as recipients or donors in transplantation were purchased from the Jackson Laboratory (Bar Harbor, ME, USA). All animal experiments were performed with the approval of the Animal Experiment Board of the Catholic University of Korea. In addition, all methods were performed in accordance with the guideline and regulations by above mentioned Board. Mice were grouped by age into neonatal (post-natal day 2) or adult groups (9–12 weeks).

### Cells and Cultures

Murine bone marrow cells were isolated by flushing whole BM. Murine hematopoietic progenitor cells were enriched by pre-treatment with 5-Fluorouracil (150 mg/kg; 5-FU, Sigma Aldrich, St Louis, MO) 4 days before BM harvest. Murine myeloid leukemic cells (MN-1) were established by retroviral transduction of MN1 into hematopoietic progenitor cells as described^43^. Briefly, progenitor-enriched, 5-FU treated cells were transduced with retroviral vector encoding MN1 and thus transformed MN1-AML cells were cultured in DMEM supplemented with 15% FBS, 20 ng/ml mouse stem cell factor (mSCF, ProSpec-Tany TechnoGene Ltd, Rehovort, Israel), 10 ng/ml human Interleukin-6 (hIL-6, ProSpec) and 6 ng/ml mouse Interleukin-3 (mIL-3, R&D Systems, Minneapolis, MN).

For co-culture with Jagged-1 inhibited MSCs, MSCs were transfected with siRNA against Jagged-1 or non-targeting RNA (Dharmacon, Inc., Lafayette, CO) 1 day before, and co-cultured for 5 dyas with lineage negative hematopoietic cells using Mouse hematopoietic progenitor cell enrichment kit (StemSep, StemCell Technologies Inc., Vancouver, BC, Canada) in MyeloCult M5300 (StemCell Technologies Inc.) supplemented with 100 ng/ml human Flt-3 ligand (FL, ProSpec), 100 ng/ml mSCF (ProSpec), 50 ng/ml human Thrombopoietin (hTPO, ProSpec) and 10^−6^ M Hydrocortisone (HC, StemCell Technologies Inc.).

For the myeloid leukemia colony-forming cell (CFU-L) assay, 1 × 10^5^ cells were cultured in semisolid media (MethoCult M3231, StemCell Technologies Inc.) supplemented with 3 U/ml of human erythropoietin (EPO, StemCell Technologies Inc.), 50 ng/ml of mSCF (ProSpec), 10 ng/ml of hIL-6 (ProSpec), and 10 ng/ml of mIL-3 (R&D Systems). For pre-B cell assays, 1 × 10^5^ cells were similarly cultured in semisolid media (Methocult M3630, Stem Cell Technologies Inc.) supplemented with IL-7.

### Characterization of Mesenchymal Stromal Cells

The uncultured fresh mesenchymal stromal cells in BM were analyzed for surface markers by flow cytometry using antibodies against CD45.2 (eBioscience, San Diego, CA), Ter119 (BD Pharmigen, San Diego, CA), CD31 (BD Pahrmigen), PDGFR-α (BD Pharmingen), and Sca-1 (eBioscience). To examine the expression of niche cross-talk molecules in mesenchymal stroma, BMCs were permeabilized and intracellular stained with specific antibodies against Jagged-1 (28H8, Cell signaling, Danvers, MA) or CXCL-12 (79018, R&D Systems) and analyzed by flow cytometry after gating for mesenchymal populations as described^12^. Relative expression levels in mesenchymal stromal populations were determined by ∆MFI, the difference in mean fluorescent intensity.

For colony-forming unit fibroblasts (CFU-Fs), 5 × 10^6^ BMCs were plated in 95 mm dishes with DMEM supplemented with 10% FBS, and colonies were visualized by crystal violet staining and counted after 14 days of culture. Cell clusters, consisting of at least 50 cells, were scored as a CFU-F colony.

### *In-vivo* repopulation

Normal bone marrow cells (9–12 weeks of mice) or leukemic cells (MN1) were transplanted into BL6 mice at postnatal day 2 (neonate) or 9–12 weeks after birth (adult). Briefly, 1 × 10^7^ of normal BMCs or 8 × 10^4^ of MN1 leukemic cells were transplanted into irradiated (300 rad) recipient mice and subsequent repopulation of the BMCs or leukemic cells were assessed by measuring the proportion of CD45.1^+^ white blood cells (WBCs) at 2 weeks after transplantation.

Lineages of repopulated hematopoietic cells were analyzed by immunostaining with anti-Mac-1/Gr-1 (myeloid), anti-CD3 (T-lymphoid), and anti-B220 (B-lymphoid; BD Pharmingen). The HSC population in BM was analyzed by staining with Sca-1-PE-Cy7 (BD Pharmingen), c-Kit-APC (eBioscience), CD150-biotin (eBioscience), CD41/48-PE (BD Pharmingen), and streptavidin-BV605 (BD Pharmingen). Leukemic cell engraftment was similarly analyzed by cell surface markers together with green fluorescent protein (GFP) expression from the transduced retroviral vector. For chemical treatment of mice, DMSO (control), DAPT (sigma, 100 mg/kg) or AMD3100 (sigma, 5 mg/kg) were injected into mice by intraperitoneal injection.

### Secondary transplantation and limiting dilution analysis for leukemia initiating cells

MN1 leukemic cells engrafted in BMs of primary recipient mice (GFP^+^/CD45.1^+^) were analyzed for phenotype (Lin-, Sca-1, c-Kit) and each subpopulation (Lin+, Lin-c-Kit−, Lin-Sca-1-c-Kit+, Lin-Sca-1 + c-Kit+) was sort purified by using the Aria lll^TM^ flow cytometry cell sorter (BD, San Diego, CA).

To quantify the number of leukemic initiating cells (LICs), each sorted subpopulation of leukemic cells was transplanted into irradiated secondary recipient mice (BL6-Ly5.2) in a serially diluted dose of purified cells as previously described^60^, and mice whose WBCs in peripheral blood contained higher than 0.1% donor-derived cells were scored as positive. The frequency of leukemia-initiating cells (LICs) was calculated by Poisson statistics using ELDA software http://bioinf.wehi.edu.au/software/elda to determine the cell dose causing 37% of the negatively engrafted mice in the serial limiting dilution transplantation.

### RNA Extraction and RT-PCR

Total RNA from bone marrow was isolated with Trizol (Invitrogen, San Diego, CA). cDNA was synthesized from l µg of total RNA with superscript III (dN6) (Invitrogen). mRNA levels of IL-7R, PU1, Foxo1, and EBF1 were measured by RT-PCR using a cDNA template and the appropriate primers. RT-PCR was performed with the ProFlex™ Thermal Cycler (Applied Biosystems Foster City, CA) and Ex taq (Takara, Otsu, Japan). Relative levels of PCR products were determined after normalizing to an endogenous GAPDH control.

### Statistical Analysis

The significance of the difference was analyzed using the Student’s t-test (p < 0.05). The frequency of LIC or leukemia-propagating cells in limiting dilution analysis was determined by Poisson statistics with a 95% confidence interval to represent ±2 SEM.

## Supplementary information

Supplementary figure
