# Supplementary material for: Age-related differences in the bone marrow stem cell niche generate specialized microenvironments for the distinct regulation of normal hematopoietic and leukemia stem cells
Source: Sci Rep. 2019 Jan 30;9:1007. doi: 10.1038/s41598-018-36999-5 (PMC6353913; doi:10.1038/s41598-018-36999-5)
Supplement: Supplementary file 1 — Supplementary figure [file 41598_2018_36999_MOESM1_ESM.docx]

Supplemental Information

**Age-related differences in the bone marrow stem cell niche generate specialized microenvironments for the distinct regulation of normal hematopoietic and leukemia stem cells.**

Ga-Young Lee^1^, Seon-Yeong Jeong^1^, Hae-Ri Lee^1^, Il-Hoan Oh^1,2,*^


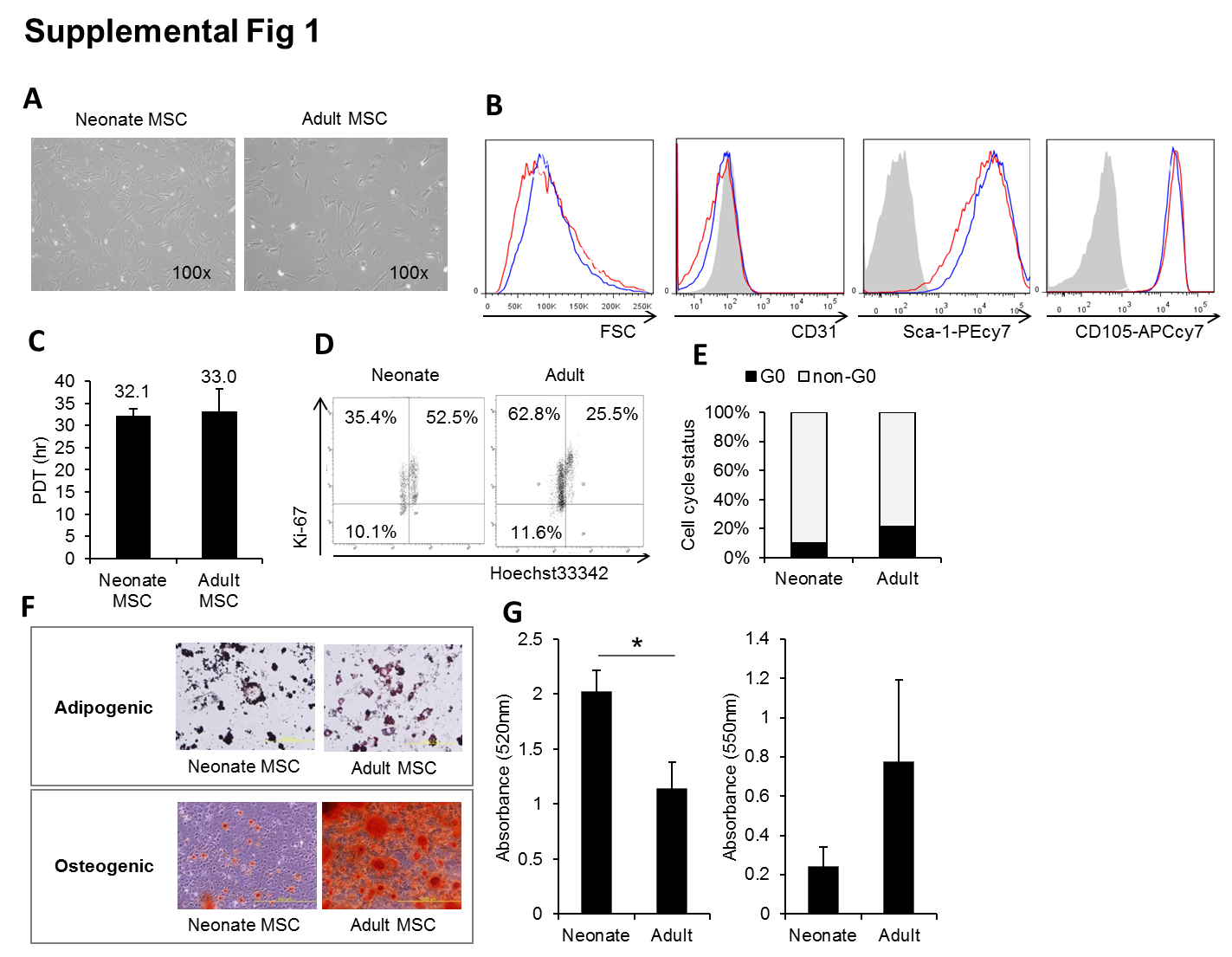


**Supplemental Figure 1. Comparisons for cultured MSCs derived from neonate and adult BM.**  MSCs were culture established from neonate (postnatal day2) or adult (9-12 weeks) BM. (A) Morphology examined by phase-contrast light microscopy (100X). (B) Flowcytometric analysis of surface makers for MSCs. Shown are the representative flowcytometry plots for each indicated surface markers. (C) Comparisons for doubling times of neonate and adult MSCs. Population doubling times (PDT) were measured by duration x log(2) / log (final cell number) - log (Initial cell number). Shown are the mean ± SEM for doubling times of each group MSCs during in-vitro culture (D, E). Cell cycle analysis of neonate and adult MSCs. MSCs were stained with Ki67 and Hoechst 33342. Shown are the representative flowcytometry plots and quantitation for cycling (G1, S/G2/M phase) and non-cycling (G0) population (mean ± SEM, n= 2, 1 expt). (F, G) Multi-lineage differentiation of neonate and adult MSCs. MSCs were induced for osteogenic and adipogenic differentiation during culture and quantified by Alizarin Red staining or lipid droplets using spectrophotometry. Shown are the mean ± SEM for absorbance at indicated wave length (n= 3, 1 expt).


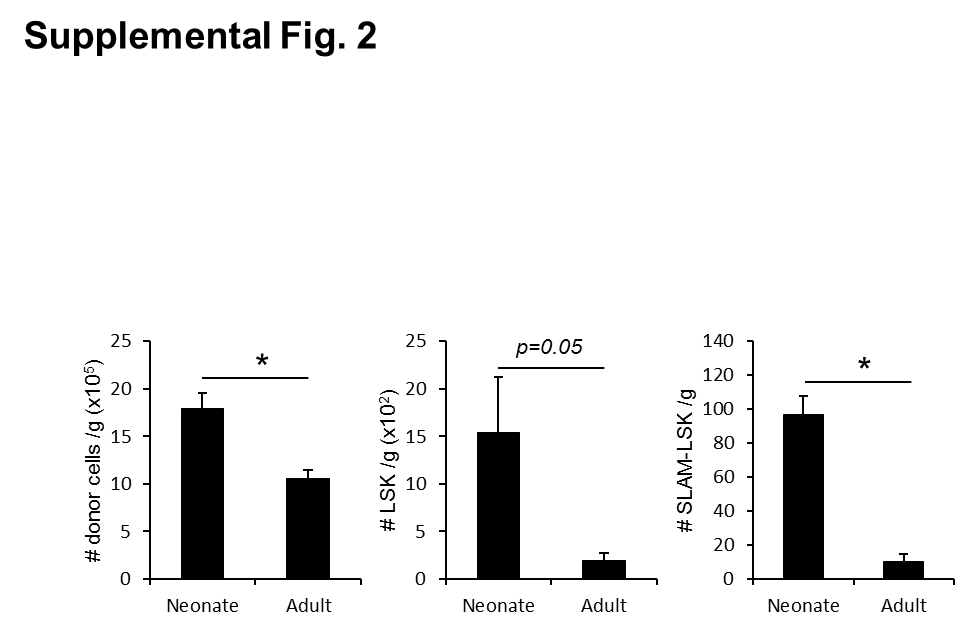


**Supplemental Figure 2. Comparisons for engraftment of donor-derived normal hematopoietic cells in neonate and adult bone marrow**. Shown are the mean ± SEM of the numbers of donor-derived cells (A), and hematopoietic stem cells (HSCs) among donor cells defined by LSK (Lin-Sca-1+c-kit+) (B) or SLAM-LSK (CD150+41-48-Lin-Sca-1+c-kit (C) after normalization by body weight (n= 4 for each group, 2 expts).


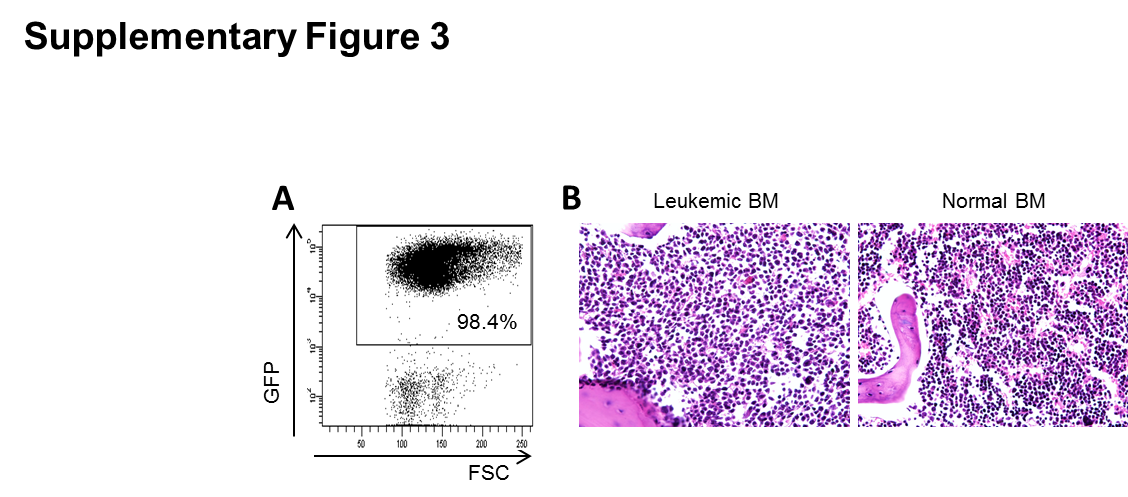


**Supplemental Figure 3. Establishment of leukemic mice model by MN1-transduced leukemic cells.**  MN1-transduced (GFP+) cells were cultured and transplanted into irradiated recipient mice. The in-vivo leukemogenesis was confirmed by flowcytometric analysis of donor leukemic cell engraftment (GFP+)(A) and histological analysis of recipient BMs (B) at magnification field (400X).


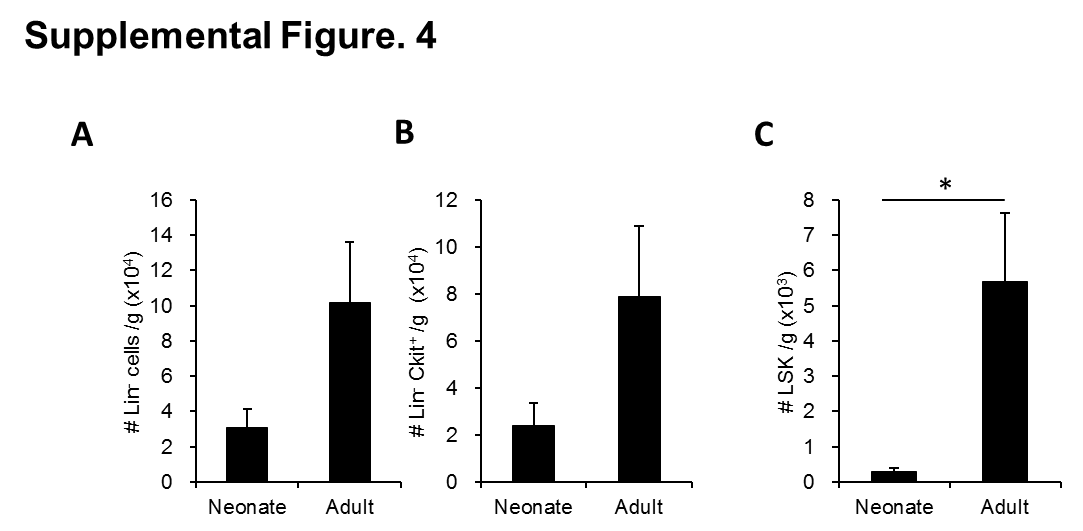


**Supplemental Figure 4. Comparisons for engraftment of leukemic cells in neonate and adult bone marrow**. Shown are the mean ± SEM of the numbers of leukemic cells in neonate and adult BM (A), and primitive leukemic cell subsets defined by LK (Lin-c-kit+) (B) or LSK (Lin-Sca-1-c-kit+) (C) after normalization by body weight (n= 13 for neonatal group, n = 6 for adult group, 5 expts).


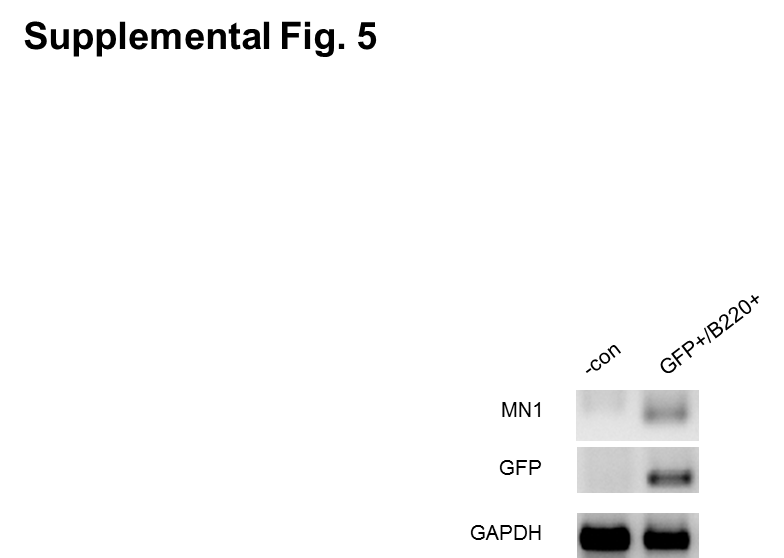


**Supplemental Figure 5. Expression of MN1 gene in B-lymphoid leukemic cells**. MN1 leukemic cells were transplanted into recipients and subsets of BM engrafted leukemic cells with B220+ phenotype were sort-purified for analysis of MN1 gene expression. Shown are representative RT-PCR plots for simultaneous expression of GFP and MN1 transcript in the sorted (GFP+/B220+) subsets.


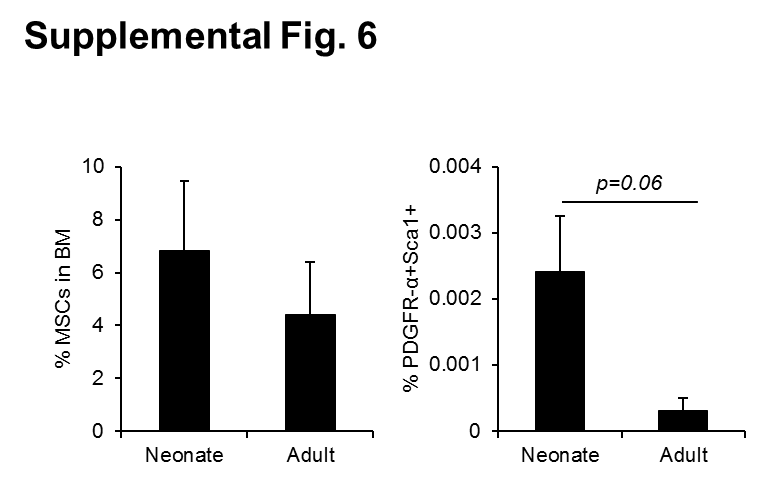


**Supplemental Figure 6. Comparisons for mesenchymal subsets in neonate and adult bone marrow after irradiation and transplantation**. Neonate and adult recipients were irradiated and transplanted with normal BM cells. Two weeks after transplantation, the recipient BMs were harvested and analyzed for mesenchymal cell population. Shown are the mean ± SEM for each indicated subsets (n= 6 for neonatal group, n = 5 for adult group, 2 expts) Note the maintenance of higher frequency of primitive subsets (PDGFR+Sca-1+) in neonate as before transplantation.
